# Nutritional Challenges in Patients with Advanced Liver Cirrhosis

**DOI:** 10.3390/jcm8111926

**Published:** 2019-11-09

**Authors:** Jessica Stirnimann, Guido Stirnimann

**Affiliations:** 1 Division of Diabetology, Endocrinology, Nutritional Medicine and Metabolism, University Hospital Inselspital and University of Bern, 3010 Bern, Switzerland; jessica.stirnimann@insel.ch; 2 University Clinic for Visceral Surgery and Medicine, University Hospital Inselspital and University of Bern, 3010 Bern, Switzerland

**Keywords:** cirrhosis, ascites, sarcopenia, sarcopenic obesity, nutrition, vitamins, micronutrients

## Abstract

Patients with advanced liver cirrhosis are at risk of malnutrition and nutrition-associated complications. Significant ascites, a frequent finding in these patients, has an especially negative impact on oral nutrition. A negative caloric and protein balance can further deteriorate the already impaired synthetic function of the cirrhotic liver. An important factor in this situation is the diminished capacity of glycogen production and storage in the cirrhotic liver and, consequently, a reduced tolerability for fasting episodes. These episodes are frequently observed in hospitalized patients, e.g., while waiting for investigations, interventions or surgery. A comprehensive work-up of patients with advanced liver cirrhosis should include not only a thorough assessment regarding nutritional deficits, but also a muscularity analysis to identify patients with sarcopenia. The overall nutritional treatment goal is to cover caloric deficits and assure a sufficiently high protein intake. Furthermore, vitamin and micronutrient deficiencies should be identified and corrective measures implemented where required. Ideally, optimal nutrition management can not only prevent the progression of malnutrition and sarcopenia in patients with advanced liver cirrhosis, but positively influence the evolution of the liver disease.

## 1. Introduction

Liver cirrhosis is a common end stage of several liver diseases. The most prevalent aetiologies are chronic hepatitis B virus (HBV) and hepatitis C virus (HCV) infection, alcoholism, and nonalcoholic steatohepatitis [1]. As, with the introduction of direct acting antiviral therapy, HCV and HCV-related complications are rapidly declining, nonalcoholic steatohepatitis is now a growing burden of cirrhosis.

In patients with advanced liver cirrhosis, malnutrition and sarcopenia are not only related to alterations in the nutritional behavior, but also to changes in the GI-tract, the liver and the muscle. An increased protein loss, via the GI-tract, the kidneys or frequent paracenteses, can further aggravate the situation.

Patients with chronic advanced liver disease suffer from increased fatigue, nausea, bloating, and anorexia, which can result in a reduced food intake. In the presence of clinically relevant ascites, mechanical effects may further compromise nutrition by compression of the stomach, resulting in early satiety [2]. Inadequate appetite regulation and energy expenditure may be related to an elevation in bound leptin in patients with cirrhosis. Whereas free leptin correlates with fat mass in cirrhotic patients as well as in healthy controls, bound leptin was positively correlated with energy expenditure in patients with cirrhosis [3]. Increased serum concentrations of bound leptin could be associated with wasting in patients with liver cirrhosis, as has been shown in patients with symptomatic human immunodeficiency virus [4]. Furthermore, leptin is also a known contributor to fibrinogenesis in chronic liver disease [5]. Small bowel transit time is correlated with the severity of liver disease, and patients with decompensated cirrhosis or spontaneous bacterial peritonitis in the context of ascites have a slower transit time [6].

In patients with alcoholic cirrhosis, anorexia and irregular unbalanced nutrition uptake are common, and energy supply may be achieved through the consumption of alcohol rather than by a balanced food intake [7]. A low socioeconomic status is an additional risk factor for poor nutrition [2].

Dietary restrictions, e.g., an untasty, sodium-reduced diet, inadequate protein supply, or taste alterations, may further compromise the nutrition of patients with cirrhosis [2].

For the assessment of malnutrition and sarcopenia different tools, comprising clinical and laboratory parameters, physical assessments, image analysis and anthropometric measurements, are available. Clinical bedside assessments are easy to perform and inexpensive, but may lack precision in patients with advanced cirrhosis, especially if significant ascites is present. In contrast, muscularity analysis on cross-sectional images is precise but technically demanding and may require special software tools and trained personnel.

## 2. Comprehensive Assessment of Patients with Advanced Liver Cirrhosis

### 2.1. Malnutrition

To assess the nutritional status of patients, the subjective global assessment (SGA) is a well-established and widely used bedside tool. Patients are assigned to one of the three different risk categories: A (well nourished), B (mildly/moderately malnourished), and C (severely malnourished), based on five items that can be derived from the patient’s history and three items that are based on clinical examinations. SGA is an independent predictor of outcome in liver transplant recipients [8,9]. However, especially in decompensated patients with ascites or peripheral fluid accumulation, the performance of SGA is limited [10].

### 2.2. Liver Function

Unfortunately, a simple test to assess the main dimensions of liver function (synthetic function, metabolic capacity and excretion function) does not exist. In the context of malnutrition, the synthetic function of the liver is of special interest. Albumin levels are frequently tested in patients with liver disease, and albumin is part of the widely used Child–Pugh–Turcotte (CPT) score to determine the stage of liver disease [11]. However, albumin levels do not solely reflect endogenous albumin production, but may also be low due to infection/inflammation, or an increased albumin loss or high in the case of albumin substitution, for instance, in patients that frequently require large volume paracenteses. In recent years, functional aspects, like the binding of lipopolisaccharides or other bacterial products and qualitative alterations (oxidation and glycation) of albumin, have been investigated in more detail. However, to date, it is not clear what the impact of these findings is [12].

Prealbumin (transthyretin) is an alternative parameter to assess the synthetic function in patients with liver cirrhosis. Prealbumin plasma levels decrease progressively from CPT stage A to C and correlate with galactose elimination capacity, a test to assess functional liver capacity [13]. A major advantage of this parameter compared to albumin is the fact that it is not influenced by exogenous albumin administration, a frequent treatment in patients with decompensated liver cirrhosis. The addition of prealbumin to the Model for End-stage Liver Disease (MELD) score improved outcome prediction in patients with decompensated liver cirrhosis [14], and prealbumin was a predictive factor regarding postoperative liver insufficiency and survival in CPT A patients with hepatocellular carcinoma-related surgery [15,16,17]. In clinical practice, prealbumin is an inexpensive and easy to determine functional liver parameter that helps to assess the synthetic liver function in cirrhotic patients. However, compared to albumin, the available evidence is limited in patients with liver cirrhosis.

Prothrombin time (PT) is the second parameter reflecting synthetic liver function that is an integral part of the CPT score (all coagulation factors, with the exception of factor VIII, are produced in the liver). However, production of coagulation factors may be preserved even in advanced cirrhosis and patients may still present with a normal or only slightly decreased PT in this situation. In contrast, patients with acute liver failure frequently have markedly decreased PT values.

### 2.3. Sarcopenia

Although sarcopenia is usually a visual diagnosis in malnourished patients with cirrhosis, diagnosis may be more challenging in obese patients, e.g., in patients with non-alcoholic fatty liver disease (NAFLD). To obtain an objective measure of sarcopenia, a quantitative analysis of the patient’s muscularity should be performed.

The most-validated method to date is the muscle assessment on cross-sectional CT or MR images. Several methods have been proposed, e.g., area of the psoas muscle at the level of the 4^th^ lumbar vertebra without and with normalization by height [18,19], the skeletal muscles index at the level of the 3rd lumbar vertebra (SMI) [19,20], or transversal psoas thickness at the level of the umbilicus [21]. Importantly, by using the SMI with the gender specific cut-offs (42 cm^2^/m^2^ for women and 50 cm^2^/m^2^ for men), muscle wasting was not limited to underweight patients, but could be detected in cirrhotic patients with a normal or even elevated BMI [20]. Regarding mortality risk in patients with cirrhosis, the skeletal muscle index (SMI) seems to be superior to the psoas index, especially in men with cirrhosis [22].

Special attention is required for patients with advanced liver cirrhosis who have a BMI >25 kg/m^2^. Sarcopenia in obese patients is frequently overlooked, since it is less expected than in underweight or normal-weight patients with malnutrition. It is important to note that sarcopenic obesity, as well as myosteatosis, characterized by an increased proportion of muscular fat, are independent risk factors regarding long-term mortality [23].

Apart from simple measurements, like psoas muscle diameter, the assessment of cross-sectional image analysis is technically demanding and may require special image analysis software and trained personnel. Therefore, image analysis is mainly used in the context of clinical research projects.

In clinical practice, bio impedance analysis (BIA), dual-energy X-ray absorptiometry (DEXA) and anthropometric measurements are easier to perform. However, DEXA and BIA results may be altered in patients with significant ascites and general fluid overload, limiting their use in decompensated patients [24]. Restricting DEXA to the upper limb lean mass, and therefore excluding the body parts that are most prone to fluid overload, resulted in a good sarcopenia-related prediction of mortality in men with advanced cirrhosis [25]. Mid-arm muscle circumference (MAMC) measurement is easy to perform, but its relevance in clinical practice is limited [26].

Unfortunately, the most frequently used scores in patients with cirrhosis, CPT and MELD score [27], do not contain specific information about the nutritional status of a given patient. Therefore, new scores have been developed and evaluated, with a focus on outcome, in patients awaiting liver transplantation. The MELD-psoas score includes transversal psoas muscle thickness normalized by height at the level of the umbilicus. This score was significantly associated with mortality, especially in patients with refractory ascites [21].

The addition of the skeletal muscle index (SMI), as a measure of sarcopenia, to the MELD score resulted in an improved prediction of mortality in patients with cirrhosis [20,28]. Especially in low MELD patients on a liver transplant list, the inclusion of sarcopenia resulted in a better association with mortality [21].

### 2.4. Deficiencies of Vitamins and Micronutrients

#### 2.4.1. Vitamin D

Vitamin D is synthesized in the skin from 7-dehydrocholesterol. An alternative source is food-derived vitamin D that is absorbed in the gastrointestinal tract with the help of bile acids. Vitamin D subsequently undergoes two hydroxylation steps, 25-hydroxylation in the liver and 1α-hydroxylation in the kidney. Whereas vitamin D is considered biologically inactive, 25-hydroxy(OH)-vitamin D is the main circulating metabolite. Conversion to 1,25-dihydroxy-vitamin D in the kidney increases the affinity for the vitamin D receptor (VDR).

The main purpose of vitamin D is the regulation of mineral and bone homeostasis. However, several other functions in different tissues (kidney, intestine, skin, immune cells and non-parenchymal hepatic cells) are known, too [29].

Vitamin D deficiency is common in liver cirrhosis. Whereas liver function correlates with vitamin D deficiency, aetiology of the liver disease seems to be of minor importance [30,31]. Decreased vitamin D levels are associated with an unfavorable outcome in patients with chronic advanced liver disease. The relative risk for hepatic decompensation and for mortality was 6.37 (95% confidence interval (CI) 1.75–23.2) and 4.31 (95% CI 1.38–13.5), respectively, for patients in the lowest OH-vitamin D level group, compared to patients in the highest OH-vitamin D level group [31].

#### 2.4.2. Vitamin A

The liver is the organ where most (>90%) of the body vitamin A is stored. Plasma contains only a fraction (1%) of the total amount of vitamin A. While normal and high vitamin A concentrations do not show a linear correlation with liver vitamin A reserves, low retinol plasma levels do correlate [32]. In patients with chronic liver disease, vitamin A levels are frequently reduced, and the concentration progressively decreases with the progression of liver disease. Importantly, patients with hepatocellular carcinoma have the lowest levels, independent of the stage of liver disease [33]. While, in patients with CPT A cirrhosis, severe deficiency was absent, 52.8% of Child–Pugh C patients presented with a severe vitamin A deficiency (<0.35 µmol/L) [34]. It is important to note that chronic high dose supplementation over a period of several years has been associated with relevant liver damage [35].

#### 2.4.3. Zinc

Zinc deficiency is a common finding in patients with advanced chronic liver disease. Reasons for low zinc levels are inadequate dietary intake and an increased zinc loss in the urine that is related to severe muscle catabolism, diuretic therapy in patients with cirrhosis and ascites [36], alcohol-induced impaired absorption, and changes in the protein and amino acid metabolism. Furthermore, endotoxins and cytokines (IL6) may play a role [37].

Since the liver is the key organ in the metabolism of zinc, liver disease can affect zinc levels and, vice versa, zinc deficiency can affect the liver. Reduced zinc levels can have a negative impact on several liver functions and impair the regeneration capacity of the liver. However, independent of the stage of cirrhosis, zinc stores in in liver, bone and muscle can be replenished over the course of six or more months [37]. In patients with cirrhosis, supplementation of zinc in combination with branched-chain amino acids (BCAA) for six months with [38] and without [39] hepatic encephalopathy has demonstrated a positive effect on blood ammonia levels. However, an increase in albumin could be demonstrated only in one of the two studies [38].

#### 2.4.4. Selenium

The essential micronutrient selenium is incorporated in at least 25 selenoproteins. Deficiency of selenium can be found in some patients with advanced chronic liver disease. In addition to a nutritional deficit, reduced selenium levels may reflect an impaired metabolism of selenomethionine to selenide in the liver. In a study by Burk et al., selenoprotein P, a protein that is mainly formed in the liver and that transports selenium to extrahepatic tissues, was reduced in parallel with the severity of the liver disease [40].

### 2.5. Bone Disease

Osteopenia and osteoporosis are common findings in patients with cirrhosis, with a prevalence of up to 68% for osteopenia and a range from 11% to 55% for osteoporosis [41]. Consequently, fractures are common in patients with chronic liver disease, and occur in up to 40% of patients with cirrhosis [42]. The pathophysiology is complex and, in addition to an altered vitamin D and calcium metabolism, other factors may be important. Hypogonadism is associated with an increased osteoclast activity, leading to accelerated bone loss. An excess of unconjugated bilirubin negatively affects the differentiation of primary osteoblasts into their primary function. Chronic glucocorticoid therapy in patients with autoimmune hepatitis is associated with osteoporosis, and environmental factors, such as chronic alcohol consumption, smoking, low BMI, sedentary lifestyle and poor nutrition have an additional negative effect on bone turnover [43].

Screening for osteopenia and osteoporosis should be considered in all patients suffering from liver cirrhosis, and be part of the standard procedure in patients that are evaluated for liver transplantation. A timely diagnosis is important to prevent fractures as typical osteoporosis-related complications. Bone mineral density screening should be repeated on a yearly basis in patients with cholestatic liver disease and/or multiple risk factors and every two to three years in other patients with liver cirrhosis [41].

## 3. Therapeutic Approach to Treat Nutritional Deficits

### 3.1. Caloric, Protein and Lipid Supplementation

In patients with advanced cirrhosis, nutrition is of paramount importance. A nutritional assessment should be performed on a regular basis and corrective measures implemented as soon as a deficit is observed. If available, a clinical nutrition specialist should perform the nutritional assessment. The aim is to identify daily deficits and to define daily nutritional requirements. This assessment should include caloric and protein intake as well as vitamins and micronutrients.

In patients with cirrhosis, a daily intake of 30–35 kcal and of 1.2 to 1.5 g protein per kg body weight (BW) is recommended [44,45]. In obese patients with liver cirrhosis, a moderately hypocaloric diet (−500–800 kcal/d), respectively a reduced daily caloric intake of 20–25 kcal/kg BW, should be combined with an increase in protein intake (>1.5 g, up to 2.5 g/kg BW) [44,45,46], Table 1.

Regarding lipids, no specific recommendations are provided in the guidelines for patients with liver cirrhosis [44,45]. Compared to the glucose and protein metabolism, alterations in the lipid metabolism seem to be of minor importance in patients with liver cirrhosis. This is illustrated by the finding that the elimination of lipid emulsions containing long-chain or long-chain and medium-chain triglycerides, and the release of free fatty acids thereof were not altered in patients with chronic hepatic failure compared to healthy controls [47,48].

Ideally, the nutritional goals can be achieved by regular meals, if required, by reducing the size and increasing the frequency of meals. A late evening snack is recommended to compensate for the reduced glycogen production and storage, and to prevent muscle proteolysis in patients with advanced cirrhosis [49]. The snack should comprise at least 50 g of complex carbohydrates [46]. If required, protein-enriched oral drinks can be added.

In patients that cannot achieve the nutritional goals with oral substitution, placement of an enteral tube should be considered. The presence of non-bleeding esophageal varices is not a contraindication for enteral tube feeding [44,45]. In contrast, a percutaneous enteral gastrostomy (PEG) is associated with an elevated risk of complications, especially bleeding, and is therefore not recommended in patients with chronic advanced liver disease [44,45].

**Figure 1:**
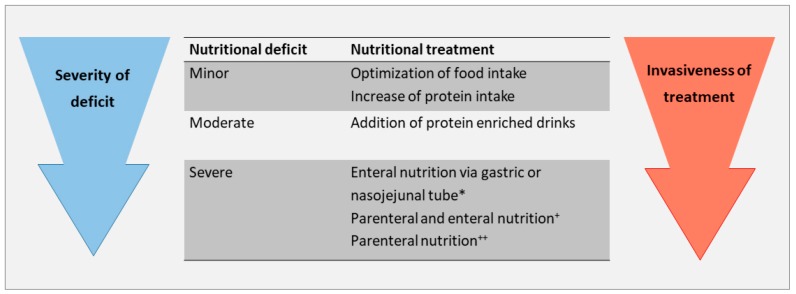
Nutrition management in malnourished patients with liver cirrhosis. * Non-bleeding esophageal varices are not a contraindication for enteral tube feeding, ^+^ if enteral nutrition is insufficient, ^++^ if enteral nutrition is not possible.

Periods of prolonged fasting should be avoided whenever possible. Risk situations are prolonged waiting time for interventions or surgery, and situations in which a patient is temporarily not able to maintain oral nutrition, e.g., during a stay in the intensive care unit. If oral or enteral feeding is not possible, parenteral nutrition should be considered early [44,45], Figure 1.

In patients with liver cirrhosis, restriction of protein intake is not indicated [44,45], since this promotes muscle proteolysis [50] and increased the risk of mortality on the waiting list for liver transplants [51]. Intolerance of mixed proteins with consecutive development of hepatic encephalopathy is very rare. In this specific situation, a diet with vegetable proteins may be beneficial [44], since vegetable proteins are rich in BCAA compared to animal proteins, and BCAA remove one mole of ammonia per mole of BCAA [52]. Alternatively, BCAA or leucine-enriched BCAA supplementation can be considered, the latter showing a stimulatory, and therefore beneficial, effect on mTORC1 in the skeletal muscle of patients with alcoholic liver cirrhosis [53]. After an episode of overt hepatic encephalopathy, BCAA supplementation improved muscle mass and minimal HE, but did not decrease recurrence of overt hepatic encephalopathy [45,54].

### 3.2. Albumin

From a nutritionist point of view, there is no indication for the substitution of albumin in patients with liver cirrhosis. However, albumin is used in patients with refractory ascites to prevent the circulatory dysfunction syndrome following paracentesis, in the setting of hepatorenal syndrome and in case of infection, especially spontaneous bacterial peritonitis (SBP) [55]. Whether long-term albumin infusions have a significant effect on morbidity and mortality in patients with advanced liver cirrhosis is currently being debated. In the ANSWER study, patients receiving albumin on a weekly basis had a lower mortality and complication rate than patients in the placebo group [56]. However, there were some concerns regarding the methodology of this trial (open label study, different management of the two treatment arms). No effect on the complication rate in patients with cirrhosis awaiting liver transplantation was found in the MACHT trial, that investigated the addition of midodrine and albumin to the standard of care treatment [57].

### 3.3. Vitamins and Micronutrients

Vitamin D has a broad impact not only on bone metabolism, but also on several hepatic cells and functions. Therefore, supplementation in case of reduced levels (<20 ng/mL) is indicated to prevent the risk of fractures that is increased in patients with cirrhosis [42], and to maintain Vitamin D related liver functions. A level of >30 ng/mL should be the therapeutic target [58,59].

For vitamin A, there is no generally accepted opinion regarding supplementation. Due to the negative effect on the liver in case of long-term high-dose substitution (>40,000 IU per day), some authors recommend against supplementation of vitamin A [34]. Taking into account the linear relationship between low serum retinol and vitamin A storage, and the association of HCC with very low vitamin A levels [33], low level substitution for a limited period of time in case of reduced vitamin A levels can be considered.

Zinc deficiency is frequent in patients with chronic liver disease and has been associated with insulin resistance, hepatic steatosis, iron overload and hepatic encephalopathy. Consequently, zinc levels should be measured in patients with cirrhosis and zinc supplemented in case of reduced levels [37,60].

Since cirrhosis causes functional selenium deficit in some patients, and, taking into account that the prevalence of selenium deficit rises with the severity of cirrhosis, supplementation in case of a confirmed deficit can be considered. However, to date, data regarding positive or negative effects of selenium substitution in patients with advanced liver cirrhosis are missing and, consequently, no general recommendation is possible. If selenium is supplemented, selenite and not selenomethionine should be prescribed, due to the altered metabolism in patients with cirrhosis [61].

### 3.4. General Management

Patients with malnutrition and sarcopenia should be followed on a regular basis by a clinical nutrition specialist, and nutritional status as well as measures to correct malnutrition and sarcopenia should be periodically reassessed. Furthermore, nutritional aspects should be integrated in the assessment and the clinical decision-making process by the multidisciplinary medical team treating patients with advanced chronic liver disease.

## 4. Conclusions

Patients with advanced liver cirrhosis are prone to malnutrition. Reasons for malnutrition are a reduced intake of food and especially proteins, a decreased synthetic function of the liver, impaired glycogen production and storage, proteolysis in the muscle, and loss of proteins via paracenteses, the kidneys or the GI tract.

A comprehensive management of patients with advanced chronic liver disease should aim at the identification of malnutrition in general and selective nutritional deficits in particular. Due to ascites and general fluid overload, body weight and body mass index may be misleading in cirrhotic patients, and a more in-depth analysis of body composition and function is indicated. SGA is an easy to perform nutritional assessment in patients with liver cirrhosis. However, in overweight and obese patients, its performance is limited, due to a weak concordance with sarcopenia [10].

Sarcopenia, a common complication in patients with advanced liver cirrhosis, is at least partially the consequence of malnutrition. Ideally, it can be diagnosed from cross-sectional abdominal images. Sarcopenic obese patients need special attention, since nutritional and muscularity deficits may be overlooked.

Treatment of patients with advanced liver cirrhosis should be comprehensive and address the individual patients’ caloric and protein requirements, as well as the correction of vitamin and micronutrient deficits. Whether continuous administration of albumin is helpful in malnourished cirrhotic patients is currently not clear, since study results are discordant.

There is certainly a need for more randomized studies, to better assess the effect of a comprehensive nutritional management on mortality and morbidity, and, in particular, on the evolution of sarcopenia and other cirrhosis-associated complications.

## Figures and Tables

**Table 1:** Nutrition management in patients with advanced liver cirrhosis.

	Cirrhosis with Malnutrition	Cirrhosis with Sarcopenic Obesity
Caloric intake	30–35 kcal/kg BW	20–25 kcal/kg BW
Protein intake	1.2 to 1.5 g/kg BW	>1.5 g, up to 2.5 g/kg BW
Lipid intake	No specific recommendation	No specific recommendation
Vitamins		
Vitamin A *	Supplement if decreased	Supplement if decreased
Vitamin D	Supplement if decreased	Supplement if decreased
Micronutrients		
Zinc	Supplement if decreased	Supplement if decreased
Selenium	Supplement if decreased	Supplement if decreased

BW: body weight. * avoid oversubstitution.
